# Serum levels of PDGF-CC as a potential biomarker for the diagnosis of Kawasaki disease

**DOI:** 10.1186/s13052-024-01580-6

**Published:** 2024-01-25

**Authors:** Jing Zhang, Penghui Yang, Yihao Liu, Zhuo Chen, Jinhui Wu, Siqi Feng, Qijian Yi

**Affiliations:** Department of Cardiovascular Medicine, Ministry of Education Key Laboratory of Child Development and Disorders, Chongqing Key Laboratory of Pediatric Metabolism and Inflammatory Diseases, National Clinical Research Center for Child Health and Disorders, National Clinical Key Cardiovascular Specialty, Children’s Hospital of Chongqing Medical University, 400014 Chongqing, China

**Keywords:** Kawasaki disease, Platelet-derived growth factor CC, Biomarker, Diagnostic markers

## Abstract

**Background:**

Kawasaki disease (KD) is an acute systemic vasculitis of unknown etiology that predominantly affects children, and no specific diagnostic biomarkers for KD are available. Platelet-derived growth factor CC (PDGF-CC) is a peptide with angiogenic properties that has been amply demonstrated to play a critical role in the cardiovascular system. This study aimed to investigate the serum expression of PDGF-CC in children with KD and to evaluate the ability of PDGF-CC to diagnose KD.

**Methods:**

A total of 96 subjects, including 59 KD patients, 17 febrile controls (FC), and 20 healthy controls (HC), were enrolled. Serum levels of PDGF-CC were measured via enzyme-linked immunosorbent assay. The associations between PDGF-CC and clinical laboratory parameters were investigated by correlation analysis. The diagnostic performance was assessed by receiver operating characteristic (ROC) curve analysis.

**Results:**

Serum PDGF-CC levels in the KD group were significantly higher than in the FC and HC groups. Serum PDGF-CC levels in the KD group were positively correlated with white blood cell counts, percentage of neutrophils, IL-2, IL-12p70, TNF-α, and IL-1β levels, and negatively correlated with the percentage of lymphocytes. In the analysis of ROC curves, the area under the curve was 0.796 (95% confidence interval 0.688–0.880; *P* < 0.0001) for PDGF-CC and increased to 0.900 (95% confidence interval 0.808–0.957; *P* < 0.0001) in combination with white blood cell counts and C-reactive protein.

**Conclusions:**

PDGF-CC is a potential biomarker for KD diagnosis, and the combination with white blood cell counts and C-reactive protein can further improve diagnostic performance.

**Supplementary Information:**

The online version contains supplementary material available at 10.1186/s13052-024-01580-6.

## Introduction

Kawasaki disease (KD), also described as mucocutaneous lymph node syndrome, is an acute self-limited systemic vasculitis that mainly occurs in children under 5 years old [1]. It is the leading cause of pediatric-acquired heart disease in developed countries, yet its etiology and mechanism of origin remain unknown. Coronary artery lesions (CAL) occur in 20–25% of untreated KD patients and may predispose to long-term cardiovascular sequels, including myocardial infarction and sudden death [2]. Timely initiation treatment with intravenous immunoglobulin (IVIG) can reduce the incidence of CAL to fewer than 5% [3], highlighting the importance of rapid diagnosis of KD. Currently, due to the absence of specific diagnostic biomarkers, the diagnostic criteria used for KD still primarily rely on clinical manifestations [4]. However, KD may share considerably similar manifestations to other pediatric febrile diseases, which may result in misdiagnosis and missed diagnosis [5]. Therefore, it is essential to search for potential biomarkers for KD diagnosis.

Platelet-derived growth factor CC (PDGF-CC) belongs to the PDGF family and is located on human chromosome 4 [6]. PDGF-CC is abundantly expressed in heart, vascular smooth muscle cells, endothelial cells, macrophages in lesions, liver, kidney, pancreas, and ovary [7–9]. PDGF-CC contains two specific protein domains: a C-terminal PDGF/VEGF core domain and an N-terminus CUB (complement subcomponents Clr/Cls, urchin EGF-like protein, and bone morphogenic protein 1) domain [6]. PDGFR-αα and PDGFR-αβ are receptors for PDGF-CC, whereas PDGFR-αα is highly expressed in endothelial cells and macrophages only under proinflammatory cytokines like interleukin 1β (IL-1β) and transforming growth factor β (TGF-β) stimulation [10]. PDGF-CC preferentially binds to and signals through PDGFR-αα involved in multiple biological processes such as atherosclerosis, diabetic cardiovascular disease, myocardial infarction, lipid metabolism, and angiogenesis, suggesting that PDGF-CC plays an essential role in the progression of vascular lesions [11–14]. In particular, PDGF-CC is reported to play important roles in atherosclerosis by stimulating the expression of matrix metalloproteinase (MMP)-9 and influencing monocyte migration and invasion in a concentration-dependent manner [11]. Additionally, cytokines associated with inflammation, such as tumor necrosis factor α (TNF-α), IL-1β, and interferon γ (IFN-γ), induced PDGF-CC mRNA expression in endothelial cells [11]. KD is a systemic vasculitis with elevated TNF-α, IL-1β, IL-6, TGF-β, MMP-9, and complement subcomponents Clr/Cls [15, 16]. Recently, several studies have shown that PDGF is associated with different forms of vasculitis [17–19]. However, related research about the relationship between PDGF-CC and KD has not yet been reported. Therefore, we examined serum PDGF-CC levels during the acute phase of KD and determined the ability of PDGF-CC to diagnose KD.

## Methods

### Subjects and general characteristics

For this study, all the serum samples were acquired from the Children’s Hospital of Chongqing Medical University between May 2022 and December 2022. Sample sizes were estimated based on similar studies and results from the pre-test. Fifty-nine patients with acute KD were selected, followed by 17 fever controls (FC) and 20 healthy controls (HC). This study was approved by the Ethics Committee of the Children’s Hospital of Chongqing Medical University and complied with the Declaration of Helsinki. Written informed consent was obtained from a parent and/or legal guardian of all participants.

These 59 KD patients met the diagnostic criteria proposed by the Japanese Kawasaki Disease Research Committee [20]. Exclusion criteria included: other immune diseases and congenital heart disease; previous history of KD; treatment with corticosteroid within the last 3 months; and treatment of IVIG or aspirin outside our hospital during the current course of disease. Echocardiography was performed before initial treatment in patients with KD, and z-scores of coronary arterial internal diameter adjusted by body surface area were calculated using the Kobayashi equation [21]. Patients with a z-score ≥ 2.0 were included in the KD with CAL (KD-CAL) group, while those with a z-score < 2 were included in the KD non-CAL (KD-NCAL) group [4]. These 17 FC patients had bacterial infections and were diagnosed with pneumonia or bronchopneumonia, upper respiratory infection, sepsis, and encephalitis.

Clinical laboratory parameters for KD patients were collected before initial IVIG therapy, including white blood cell counts (WBC), platelet counts (PLT), hemoglobin (HB), percentage of neutrophils (N%), percentage of lymphocytes (L%), red blood cell counts (RBC), mean platelet volume (MPV), platelet distribution width (PDW), C-reactive protein (CRP), procalcitonin (PCT), albumin (ALB), aspartate aminotransferase (AST), alanine aminotransferase (ALT), interleukin-2(IL-2), IL-4, IL-6, IL-10, IL-12p70, IL-1β, and tumor necrosis factor-α(TNF-α).

### Sample collection and measurement of serum PDGF-CC levels

Serum samples were obtained from children with KD before initial IVIG treatment and centrifuge at 1000 rpm for 20 min, then stored at -80 °C for later use. The same approach was followed to obtain serum samples from FC and HC.

The expression of PDGF-CC protein was determined by the ELISA kit according to the protocol. Briefly, 0.1 mL of diluted antibody (1 g/mL) was added to the reaction wells and placed at 4 °C overnight. The diluted sample to be tested (0.05 mL) and the newly diluted ELISA antibody (0.05 mL) are added to the reaction wells and incubated successively for 1 h at 37 °C. Then, 0.1 mL of 3,3′,5,5′-tetramethylbenzidine (TMB) substrate solution was prepared and added to each reaction well and incubated at 37 ℃ for 10–30 min. The reaction was stopped by the addition of sulfuric acid (2 M, 0.05 mL), and the OD value was measured at 450 nm using a microplate reader. The enzyme-linked immunosorbent assay (ELISA) kit was purchased from Jianglai Biological Company, China.

### Statistical analysis

Statistical analysis was performed with R (R Core Team, version 4.2.3) and GraphPad Prism 8 (GraphPad Software, Inc., San Diego, CA, USA). A bilateral *P* value of < 0.05 was considered a statistically significant difference. Normality test was performed with Shapiro-Wilk test. Descriptive statistics are presented as mean ± standard deviation, median (P25-P75), or number and percentage (n, %). Data were compared using student’s t-test (normality) or Mann–Whitney U test (non-normality). Pearson correlation was used to measure correlation. Receiver operating characteristic (ROC) curve analysis was used to evaluate the diagnostic performance, and the area under the curve (AUC), specificity, sensitivity, positive likelihood ratio (LR+), negative likelihood ratio (LR-), and Youden index were calculated.

## Results

### Clinical characteristics of patients with KD

Among 59 patients with KD, 26 were male, and 33 were female, with a median age of 31 months (age range, 20–49 months). There were no significant differences in sex and age between the KD and the FC groups. The levels of WBC, N%, CRP, PCT, and ALT in the KD group were significantly higher than those in the FC group. Conversely, patients with KD had significantly lower levels of HB, L%, and ALB compared with the FC group (Table 1).

**Table 1:** Demographic and clinical laboratory parameters in the FC and KD groups

Variable	FC (*n* = 17)	KD (*n* = 59)	*PP* value
Age (month)	30.00 (19.00–38.00)	31.00 (20.00–49.00)	0.454
Sex (male/female)	9/8	26/33	0.711
WBC (10^9^/L)	10.60 ± 3.77	13.81 ± 4.34	0.007*
PLT (10^9^/L)	344.65 ± 80.31	347.59 ± 117.41	0.923
HB (g/L)	116.35 ± 9.60	109.00 ± 11.71	0.021*
N%	50.00 (39.00–63.00)	69.50 (60.75–75.55)	< 0.001*
L%	43.00 (29.00–55.00)	22.20 (17.40–31.60)	< 0.001*
RBC (10^9^/L)	4.39 (4.03–4.58)	4.05 (3.84–4.37)	0.073
MPV (fl.)	9.50 (9.05-10.00)	9.80 (9.40-10.35)	0.100
PDW (fl.)	10.10 (9.15–10.80)	10.20 (9.55–11.65)	0.295
CRP ((mg/L)	18.00 (8.00–31.00)	48.94 (25.73–82.75)	< 0.001*
PCT (ng/mL)	0.23 (0.10–0.56)	0.56 (0.22–1.21)	0.019*
ALB (g/L)	42.00 (37.30–45.00)	38.30 (35.10-41.25)	0.002*
AST (U/L)	32.10 (27.40–37.00)	27.40 (23.85–38.55)	0.178
ALT (U/L)	15.30 (13.00-22.80)	24.50 (15.45–69.75)	0.034*

Note: FC, febrile controls; KD, Kawasaki disease; WBC, white blood cell counts; PLT, platelet counts; HB, hemoglobin; N%, percentage of neutrophils; L%, percentage of lymphocytes; RBC, red blood cell counts; MPV, mean platelet volume; PDW, platelet distribution width; CRP, C-reactive protein; PCT, procalcitonin; ALB, albumin; AST, aspartate aminotransferase; ALT, alanine aminotransferase;*P < *0.05*

### Serum levels of PDGF-CC in KD, FC, and HC

**Fig. 1:**
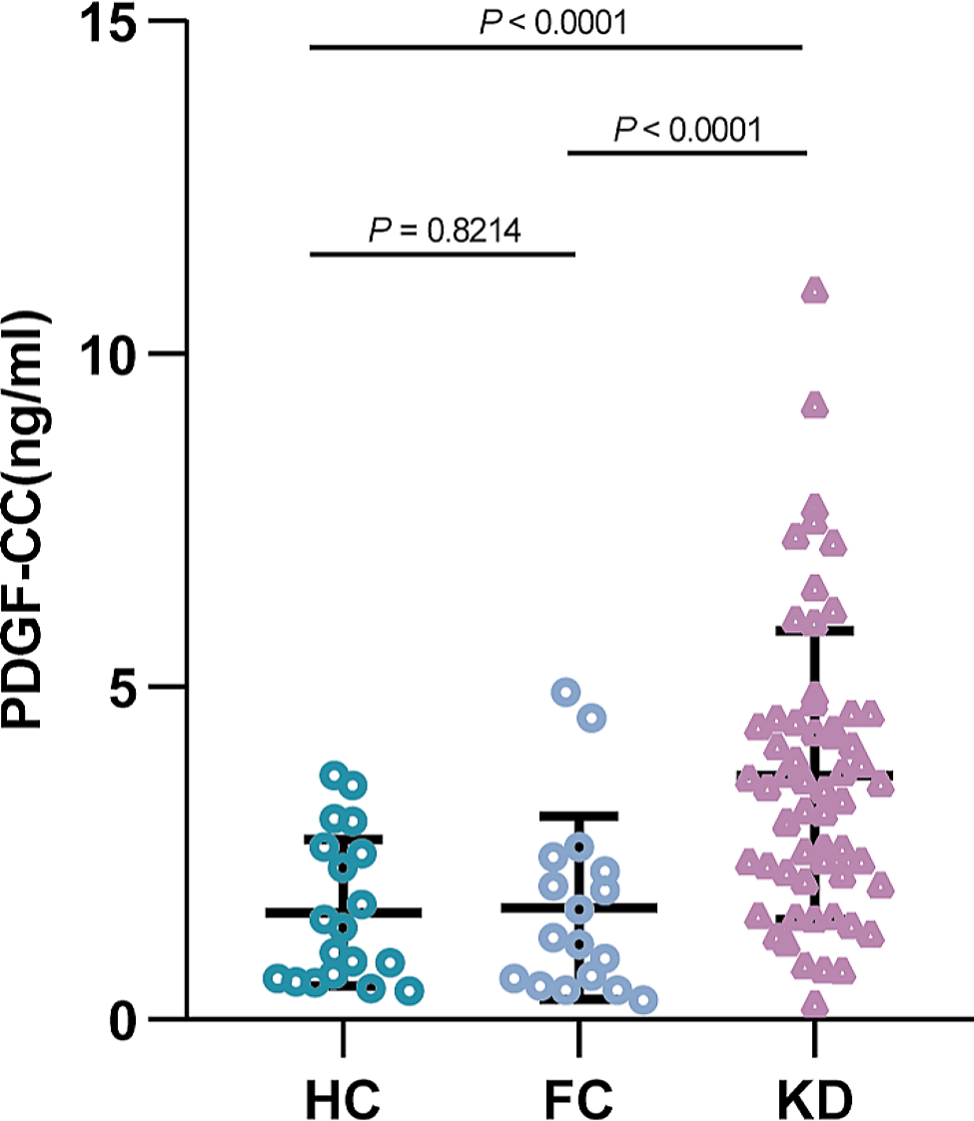
Serum PDGF-CC levels in KD, FC, and HC. PDGF-CC, Platelet-derived growth factor CC; KD, Kawasaki disease; FC, Febrile control; HC Healthy control

As shown in Fig. 1, serum PDGF-CC levels in the KD group (3.504 ng/ml) were significantly higher than those in the FC group (1.237 ng/ml) and HC (1.193 ng/ml) groups (*P* < 0.0001).

### Correlation of serum levels of PDGF-CC with the general laboratory data and inflammatory cytokines in KD

**Fig. 2:**
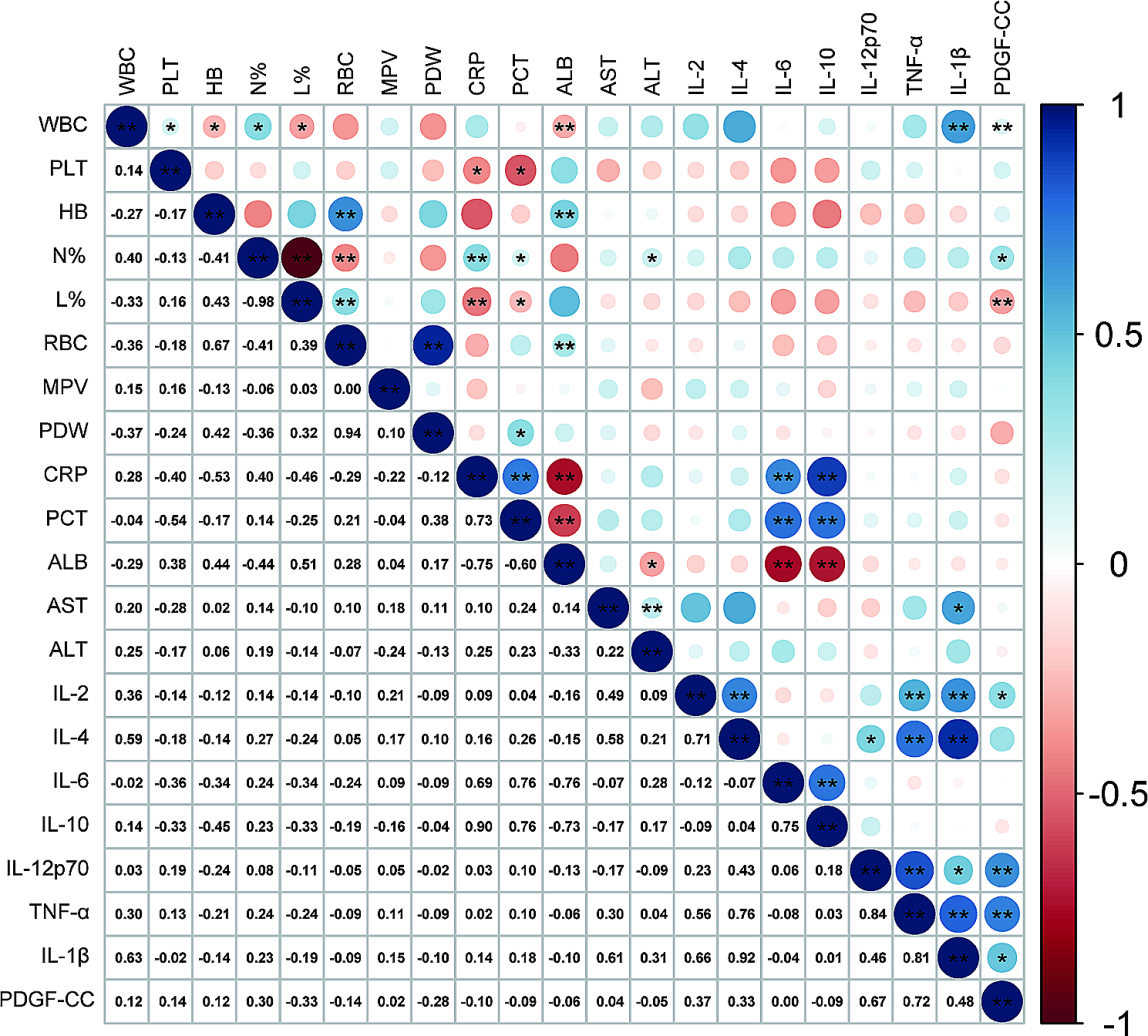
Correlation analysis between PDGF-CC and clinical parameters for KD. PDGF-CC, Platelet-derived growth factor CC; KD, Kawasaki disease; WBC, white blood cell counts; PLT, platelet counts; HB, hemoglobin; N%, percentage of neutrophils; L%, percentage of lymphocytes; RBC, red blood cell counts; MPV, mean platelet volume; PDW, platelet distribution width; CRP, C-reactive protein; PCT, procalcitonin; ALB, albumin; AST, aspartate aminotransferase; ALT, alanine aminotransferase; *, *P* < 0.05; **, *P* < 0.01

Correlation between serum levels of PDGF-CC and the general laboratory data and inflammatory cytokines in KD was performed by Pearson correlation analysis, and the result was visually displayed in a heatmap diagram (Fig. 2). We found that the expression levels of PDGF-CC were positively correlated with WBC (*r* = 0.12, *P* < 0.01), N% (*r* = 0.30, *P* < 0.05), IL-2 (*r* = 0.37, *P* < 0.05), IL-12p70 (*r* = 0.67, *P* < 0.01), TNF-α (*r* = 0.72, *P* < 0.01), and IL1-β (*r* = 0.48, *P* < 0.05), and negatively correlated with L% (*r* = − 0.33, *P* < 0.01).

### Potential diagnostic value of serum PDGF-CC for KD

**Fig. 3:**
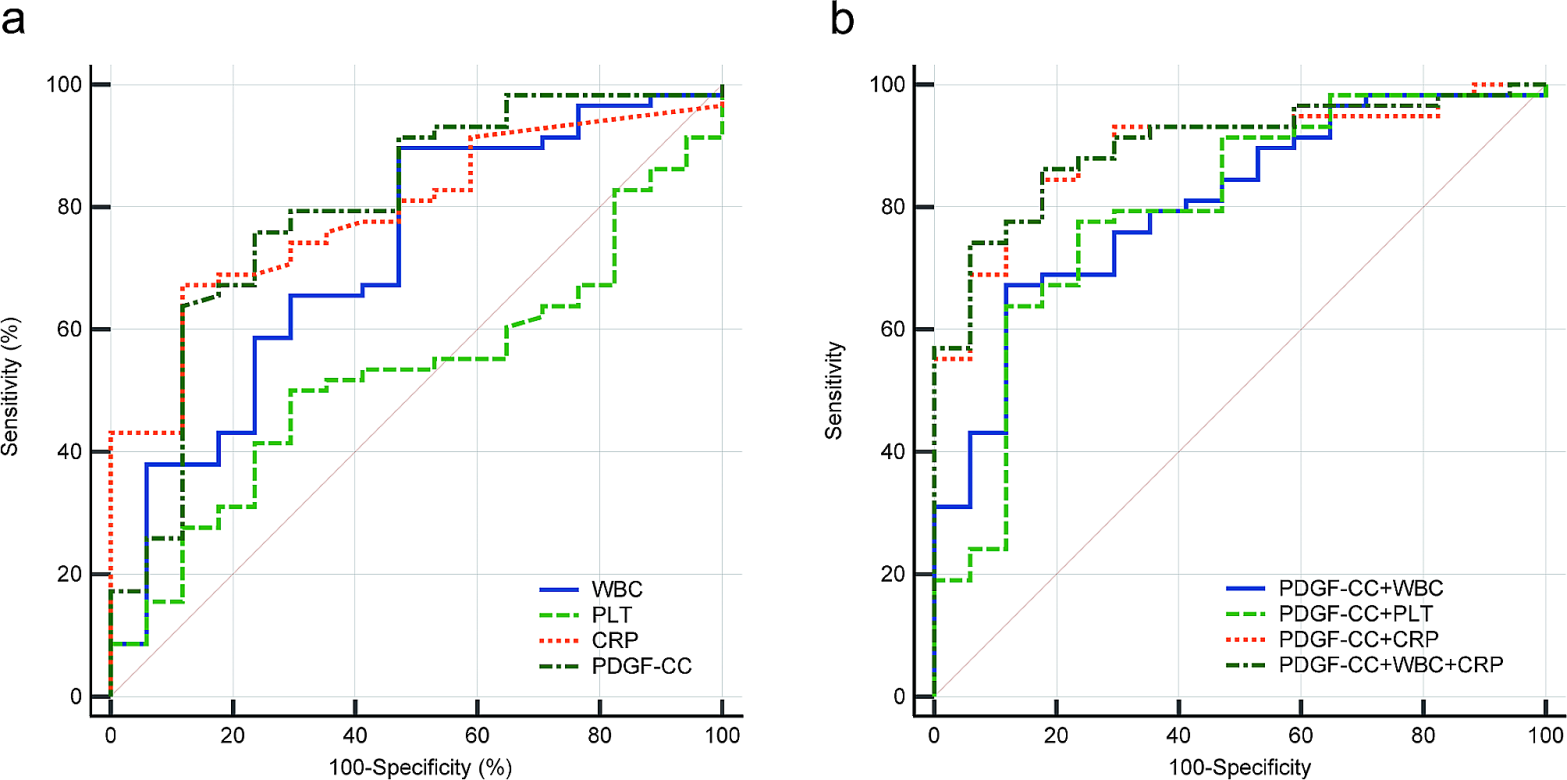
ROC curves of biomarkers to distinguish KD from FC. ROC, receiver operating characteristic; PDGF-CC, Platelet-derived growth factor CC; WBC, white blood cell counts; PLT, platelet counts; CRP, C-reactive protein

ROC curve analysis was performed to estimate the ability of PDGF-CC, WBC, PLT, and CRP to distinguish KD from FC. The results indicated that the AUC value of PDGF-CC was 0.796 (cut-off of 2.22, sensitivity of 74.58%, and specificity of 76.47%), which was higher than the AUC of other single markers (Fig. 3a; Table 2). Then, the PDGF-CC was further singly or doubly combined with the other 3 biomarkers. We found that the combination of PDGF-CC and WBC increased its AUC value from 0.796 to 0.813, the combination of PDGF-CC and CRP increased its AUC value from 0.796 to 0.892, and the combination of PDGF-CC, WBC, and CRP obtained the highest AUC of 0.900 (Fig. 3b; Table 2).

**Table 2:** Predictive ability of biomarkers to distinguish KD from FC.

	*PP*	AUC	95%CI	cut-off value	Se(%)	Sp(%)	+LR	-LR	Youden Index
WBC	0.0028*	0.720	0.605–0.817	9.87	89.83	52.94	1.91	0.19	0.428
PLT	0.7478	0.523	0.406–0.639	313.00	49.15	70.59	1.67	0.72	0.197
CRP	< 0.0001*	0.790	0.680–0.875	36.00	67.24	88.24	5.72	0.37	0.555
PDGF-CC	< 0.0001*	0.796	0.688–0.880	2.22	74.58	76.47	3.17	0.33	0.511
PDGF-CC + WBC	< 0.0001*	0.813	0.707–0.893	0.74	74.58	76.47	3.17	0.33	0.502
PDGF-CC + PLT	< 0.0001*	0.797	0.689–0.880	0.78	66.10	88.24	5.62	0.38	0.543
PDGF-CC + CRP	< 0.0001*	0.892	0.800-0.952	0.70	84.48	82.35	4.79	0.19	0.668
PDGF-CC + WBC + CRP	< 0.0001*	0.900	0.808–0.957	0.62	86.21	82.35	4.89	0.17	0.686

Note: KD, Kawasaki disease; FC, Febrile control; WBC, white blood cell counts; PLT, platelet counts; CRP, C-reactive protein; AUC Area under the curve; Se, sensitivity; Sp, specificity; +LR, positive likelihood ratio; -LR, negative likelihood ratio; *P < *0.05*

### Serum PDGF-CC and clinical laboratory parameters between the KD‑NCAL and KD‑CAL groups

As shown in Table 3, no significant differences observed in WBC, PLT, HB, N%, L%, RBC, MPV, PDW, PCT, ALB, AST, ALT, and PDGF-CC between the KD-CAL and KD-NCAL groups (*P* > 0.05), except for CRP (Table 3).

**Table 3:** PDGF-CC levels and clinical laboratory parameters in the KD-NCAL and KD-CAL groups

Variable	KD-NCAL (*n* = 33)	KD-CAL (*n* = 26)	P value
WBC (10^9^/L)	13.31 ± 4.44	14.44 ± 4.21	0.324
PLT (10^9^/L)	322.09 ± 100.83	379.96 ± 130.46	0.060
HB (g/L)	110.59 ± 11.58	107.04 ± 11.79	0.254
N%	69.50 (63.30–75.80)	69.70 (57.85–74.97)	0.909
L%	22.20 (17.50–30.00)	22.45 (15.95–35.45)	0.945
RBC (10^9^/L)	4.06 (3.86–4.42)	4.04 (3.82–4.24)	0.332
MPV (fl.)	9.80 (9.50–10.60)	9.70 (9.20-10.12)	0.209
PDW (fl.)	10.60 (9.85–11.95)	9.90 (9.47–10.75)	0.108
CRP (mg/L)	35.35 (12.17–65.89)	77.29 (46.61-107.92)	0.002*
PCT (ng/mL)	0.37 (0.20–0.91)	0.80 (0.32–1.86)	0.067
ALB (g/L)	38.65 (36.58–40.60)	37.10 (32.90-41.65)	0.219
AST (U/L)	27.80 (24.00-36.60)	25.00 (23.05–39.50)	0.581
ALT (U/L)	24.00 (15.00-59.50)	28.00 (17.00-78.15)	0.618
PDGF-CC (ng/ml)	3.39 (2.21–3.86)	3.85 (2.33–4.62)	0.275

Note: KD, Kawasaki disease; CAL, coronary artery lesion; NCAL, non- coronary artery lesion; WBC, white blood cell counts; PLT, platelet counts; HB, hemoglobin; N%, percentage of neutrophils; L%, percentage of lymphocytes; RBC, red blood cell counts; MPV, mean platelet volume; PDW, platelet distribution width; CRP, C-reactive protein; PCT, procalcitonin; ALB, albumin; AST, aspartate aminotransferase; ALT, alanine aminotransferase;*P < *0.05*

## Discussion

The PDGF family contributes strongly to multiple mechanisms in vascular pathologies such as atherosclerosis, restenosis, aortic aneurysms, pulmonary hypertension, and vasculitis [22]. A prospective longitudinal study found that the serum PDGF level of patients with recurrent giant cell arteritis was higher than those who achieved remission [23]. Another retrospective study demonstrated that PDGF can be used as a marker for the clinical diagnosis of cerebral amyloid angiopathy-related inflammation/vasculitis [18]. In line with these, imatinib mesylate, a PDGFR tyrosine kinase inhibitor, has been shown to have therapeutic potential for refractory eosinophilic granulomatosis with polyangiitis and giant cell arteritis [24, 25]. These studies emphasize the potential role of PDGF in vasculitis. PDGF-CC, as a member of PDGF family, is a potent inducer of monocytes and macrophages, can modulate monocyte-mediated inflammatory responses [26]. Previous studies have shown that monocytes and macrophages play an essential role in forming vasculitis in the acute phase of KD [27]. Furthermore, PDGF-CC is important for stimulating and maintaining endothelial function [12]. PDGF-CC has been reported to play an important role in atherosclerosis, lipid metabolism, and angiogenesis [11, 14]. However, the potential function of PDGF-CC in KD has not been investigated. In this study, we examined serum PDGF-CC concentrations in KD patients for the first time and further explored the potential role of PDGF-CC in KD.

Our study demonstrated that serum PDGF-CC levels in the KD group were significantly higher than in the HC and FC groups. In addition, correlation analysis showed that serum PDGF-CC levels were positively correlated with WBC, N%, IL-2, IL-12p70, TNF-α, and IL-1β levels and were negatively correlated with L% and TT in patients with KD. Meanwhile, the AUC value of PDGF-CC was 0.796 (with sensitivity of 74.58% and specificity of 76.47%) for differentiating between KD and FC and was elevated to 0.900 (with sensitivity of 86.21% and specificity of 82.35%) when combined with WBC and CRP. These results indicated that serum PDGF-CC may be a diagnostic biomarker for KD, and the combination of PDGF-CC, WBC, and CRP provides a better predictive value for the early diagnosis of KD.

PDGF-CC was found to be involved in many cardiovascular diseases. PDGF-CC participates in the formation of atherosclerosis by triggering MMP-2 and MMP-9 expression and monocyte migration and invasion [11]. Indeed, PDGF-CC protein treatment enhanced post-ischemic revascularization in mouse hearts with myocardial infarction by increasing vessel density and SMC coverage [14, 28]. The present study found that serum PDGF-CC levels were significantly higher in acute KD patients than in HC and FC, suggesting that PDGF-CC may be involved in vascular inflammation in the acute phase of KD. Histopathology of coronary arteries in patients with KD reveals a critical role of monocytes and macrophages in KD vasculitis. Macrophages, in particular, are key drivers of KD vasculitis, producing inflammatory cytokines such as TNF-α and VEGF and proteases such as MMP-2 and MMP-9, which may disrupt elastin and structural components within the arterial wall, leading to disruption of the vascular wall support system and eventual CAL development. Previous studies have shown that PDGF-CC plays an important role in regulating monocyte chemotaxis and stimulating the expression of MMP-9 [7]. Taken together, we speculate that PDGF-CC may play an important role in vascular inflammation in the acute phase of KD through inflammatory activation.

During the acute stage of KD, elevated WBC and neutrophil predominance are characteristic laboratory findings, and hypercytokinemia is the pathophysiological feature [4, 29]. The current study noted that serum PDGF-CC levels in KD patients were positively correlated with WBC, N%, IL-2, IL-12p70, TNF-α, and IL-1β and negatively correlated with L%, supporting the hypothesis that PDGF-CC exerts its effect in the KD vasculitis. It has been reported that TNF-α, IL-1β, and INF-γ induce PDGF-CC mRNA expression in endothelial cells [11], and the stimulation of proinflammatory cytokines such as IL-1β and TGF-β also leads to high expression of the receptor PDGFR-αα in vascular endothelial cells and macrophages [10, 30]. These cytokines are increased to induce the secretion of PDGF-CC. The pathologic process of KD is complex, many of which remain unclear. The above information indicates that serum TNF-α, IL-1β, INF-γ, and PDGF-CC may synergistically involve KD-associated vasculitis.

We further tested the potential diagnostic value of PDGF-CC in KD by using ROC curve analysis. As a single biomarker, PDGF-CC had the highest AUC of 0.796 (sensitivity: 74.58; specificity: 76.47) to distinguish KD from FC, implying that serum PDGF-CC can be used as a novel biomarker for KD. When PDGF-CC was combined with WBC and CRP, the AUC increased to 0.900, the sensitivity increased to 86.21, and the specificity increased to 82.35. The combined biomarkers enhance the predictive value for KD.

Notably, our study addressed that no statistical difference in PDGF-CC was observed regardless of CAL. Similarly, there are no significant differences in several laboratory parameters (WBC, PLT, HB, N%, ALB, AST, ALT) regardless of CAL, although these are considered predictors of CAL development [31–33]. This result may be due to the small sample size. More experiments are needed to further explore the role of PDGF-CC in KD.

There are several limitations to this study. First, this was a single-center study with a relatively small number of participants. Other multi-center research is necessary to confirm and extend the current results. Second, due to the lack of data on serum PDGF-CC levels at different time points in patients with KD, we were unable to reveal the trend of serum PDGF-CC in KD development.

## Conclusion

In conclusion, the present study demonstrated that the serum level of PDGF-CC was increased in patients with KD. Serum PDGF-CC can be a potential biomarker for KD, and its combined use of WBC and CRP can further increase diagnostic performance.

### Electronic supplementary material

Below is the link to the electronic supplementary material.

Supplementary Material 1

## Data Availability

The datasets used and/or analyzed during the current study are available from the corresponding author on reasonable request.

## Abbreviations

ALT: Alanine aminotransferase
AST: Aspartate aminotransferase
AUC: Area under the curve
CAL: Coronary artery lesion
CRP: C-reactive protein
ELISA: Enzyme-linked immunosorbent assay HB,hemoglobin
IL-10: Interleukin-10
IL-12p70: Interleukin-12p20
IL-1β: Interleukin-1β
IL-2: Interleukin-2
IL-4: Interleukin-4
IL-6: Interleukin-6
KD: Kawasaki disease
L%: Percentage of lymphocytes
MMP-2: Metalloproteinase-2
MMP-9: Metalloproteinase-9
MPV: Mean platelet volume
N%: Percentage of neutrophils
NCAL: Non-coronary artery lesion
PCT: Procalcitonin
PDW: Platelet distribution width
PLT: Platelet counts
RBC: Red blood cell counts
ROC: Receiver operating characteristic
Se: Sensitivity
Sp: Specificity
TNF-α: Tumor necrosis factor-α
WBC: White blood cell counts
+LR: Positive likelihood ratio
-LR: Negative likelihood ratio
