# Supplementary material for: Serum levels of PDGF-CC as a potential biomarker for the diagnosis of Kawasaki disease
Source: Ital J Pediatr. 2024 Jan 25;50:16. doi: 10.1186/s13052-024-01580-6 (PMC10809580; doi:10.1186/s13052-024-01580-6)
Supplement: Supplementary file 1 — Supplementary Material 1 [file 13052_2024_1580_MOESM1_ESM.docx]

**Response Letter to “Italian Journal of Pediatrics” Submission**

Manuscript ID: ITJP-D-23-00679.

Manuscript Title: Serum levels of PDGF-CC as a potential biomarker for the diagnosis of Kawasaki disease.

**Reply to the Editor**

Dear Dr. Elena Bozzola,

On behalf of my co-authors, thank you for providing us with this great opportunity to submit a revised version of our manuscript. We appreciate the reviewers for their insightful and constructive feedback. We have considered the comments very carefully and have revised the manuscript accordingly.

We hope this revised manuscript has addressed your and the reviewers' concerns and look forward to hearing from you.

Sincerely,

Qijian Yi

Encl. Responses to the comments from Reviewer 1, 2, and 3.

**Reply to Reviewer #1**

Dear Reviewers,

Thank you very much for your time reviewing our manuscript and for your valuable comments and suggestions.

**Comment:**

Excellent article, very up-to-date and highly relevant, well written, acceptable English, with clear and simple objectives, relevant conclusions for the pediatric field, just check reference 15 please. Statistical treatment according to well-used ROC and AUC diagnostic tests, excellent graphs. Objective conclusions. Very interesting

**Response:**

Thanks for your great suggestion on improving our manuscript. We have removed the reference 15 from the revised manuscript.

We hope you will be satisfied with this revised version.

Sincerely,

Qijian Yi

-----End of Reply to Reviewer #1------

**Reply to Reviewer #2**

Dear Reviewers,

Thanks a lot for your time involved in reviewing our manuscript and your valuable feedback. We also appreciate your detailed comments and hope our explanation has fully addressed all your concerns. In the remainder of this letter, we discuss each of your comments individually, along with our corresponding responses. To facilitate this discussion, we first retype your comments and then present our responses to the comments.

**Comment 1:**

The introduction should include at least one reference about the relationship between PDGF and vasculitis in general.

**Response 1:**

We added three references on PDGF and vasculitis to the introduction: giant cell arteritis, cerebral amyloid angiopathy-related inflammation/vasculitis, and Takayasu’s arteritis.

We have added the above content to the revised manuscript (paragraph 1, page 4). The relevant contents are provided below for your quick reference.

Recently, several studies have shown that PDGF is associated with different forms of vasculitis[17–19]. However, related research about the relationship between PDGF-CC and KD has not yet been reported.

Ref 17. Kaiser M, et al. Platelet-derived growth factor, intimal hyperplasia, and ischemic complications in giant cell arteritis. Arthritis Rheum. 1998;41:623–33.

Ref 18. Sakai K, et al. Cerebrospinal fluid cytokines and metalloproteinases in cerebral amyloid angiopathy-related inflammation. Acta Neurol Scand. 2021;143:450–7.

Ref 19. Arnaud L, et al. Pathogenesis of Takayasu’s arteritis: a 2011 update. Autoimmun Rev. 2011;11:61–7.

**Comment 2:**

Study design should be written clearly in the beginning of this section.

**Response 2:**

We have rewritten the beginning of the Methods section to present our study design clearly.

We have added the above content to the revised manuscript (paragraph 2, page 4). The relevant contents are provided below for your quick reference.

For this study, all the serum samples were acquired from the Children's Hospital of Chongqing Medical University between May 2022 and December 2022. Sample sizes were estimated based on similar studies and results from the pre-test. Fifty-nine patients with acute KD were selected, followed by 17 fever controls (FC) and 20 healthy controls (HC). This study was approved by the Ethics Committee of the Children's Hospital of Chongqing Medical University and complied with the Declaration of Helsinki. Written informed consent was obtained from a parent and/or legal guardian of all participants.

**Comment 3:**

The basics of sample size calculation is missing.

**Response 3:**

The sample size of our study was calculated according to the sample size from similar studies and results from the pre-test. First, our review of the literature revealed that previous studies on biomarkers and KD often had small sample sizes. For example, in Zeng L et al.’s study, 70 KD, 20 FC, and 31 HC were included. Jakob A et al. included 31 KD and 20 HC. Wang Z et al. included 40KD, 20FC, and 20HC. Second, we calculate the sample size based on independent sample mean analysis. We considered the estimated ratio of sample sizes between the KD and FC groups from the pre-test and the mean and standard deviation of PDGF-CC for both groups. With a two-sided α level of 0.05 and a power of 80%, we calculated the estimated sample size based on these factors. The results of the estimated sample size were 39 KD and 13 FC. Finally, we enrolled 59 KD and 17 FC in our study. Post hoc power analysis revealed that the statistical power value was 0.91 for our sample size. In general, we believe this sample size is sufficient for our research. In addition, we added the sentence “Sample sizes were estimated based on similar studies and results from the pre-test” to the beginning of the Methods section.

(Ref.

Zeng L, et al. Prokineticin 2 as a potential biomarker for the diagnosis of Kawasaki disease. Clin Exp Med. 2023 Nov;23(7):3443-3451.

Jakob A, et al. Kawasaki disease and increased cardiovascular risk: Is there a link to circulating glycocalyx biomarkers? Microvasc Res. 2022 Mar;140:104269.

Wang Z, et al. The diagnostic role of AIM2 in Kawasaki disease. Clin Exp Med. 2021 Feb;21(1):41-47. doi: 10.1007/s10238-020-00669-6. Epub 2020 Oct 20.)

**Comment 4:**

Number of KD children should be mentioned here.

**Response 4:**

We have reported the number of KD patients in this revised sentence – “These 59 KD patients met the diagnostic criteria proposed by the Japanese Kawasaki Disease Research Committee”.

**Comment 5:**

- The exclusion criteria were as follows: (1) with other immune diseases; (2) with a previous diagnosis of KD; (3) treatment with corticosteroid within the last 3 months; (4) with out-of-hospital use of IVIG or aspirin during the current course of the disease; (5) with congenital heart disease.

-This part should be rewritten in better way with better and clearer English.

**Response 5:**

We have rewritten the exclusion criteria in better and clearer English.

We have added the above content to the revised manuscript (paragraph 3, page 4). The relevant contents are provided below for your quick reference.

Exclusion criteria included: other immune diseases and congenital heart disease; previous history of KD; treatment with corticosteroid within the last 3 months; and treatment of IVIG or aspirin outside our hospital during the current course of disease.

**Comment 6:**

Why the number of controls were less than the number of cases and why febrile children were included as control and what type of febrile illnesses were they?

**Response 6:**

The sample size of our study was calculated according to the sample size from similar studies and results from the pre-test. As we stated in **Response 3**, the number of controls in this study is sufficient to support our analysis and conclusions.

Because KD may share considerably similar manifestations to other pediatric febrile diseases, which may result in misdiagnosis and missed diagnosis, the early prediction of KD, especially in distinguishing KD from other FC, is a focus of KD research. Therefore, febrile children were included as controls in the current study. The febrile illnesses include pneumonia or bronchopneumonia, upper respiratory infection, sepsis, and encephalitis.

We have added the above content to the revised manuscript (paragraph 3, page 4). The relevant contents are provided below for your quick reference.

These 17 FC patients had bacterial infections and were diagnosed with pneumonia or bronchopneumonia, upper respiratory infection, sepsis, and encephalitis.

**Comment 7:**

-Data were compared using student's t-test (normality) or Mann–Whitney U test (non-normality).

-Why both parametric and non-parametric tests were used on the same sample and was there any test of normality performed on the data in the beginning of the statistical analysis?

**Response 7:**

The normality results of different variables of the same sample may be different. For example, in this study, in the comparison of KD and FC groups, WBC, PLT, and HB were normally distributed, while Age, N%, L%, RBC, MPV, PDW, CRP, PCT, ALB, AST, ALT were non-normally distributed. Normally distributed data are presented as the mean ± standard deviation, and non-normally distributed data are presented as the median (P25-P75), which have been presented in Table 1. Further, the student's t-test was used for normally distributed data, and the Mann-Whitney U test was used for non-normally distributed data. Yes, we carried out the normality test prior to the statistical analysis. We have added this information to the Statistical analysis section – “Normality test was performed with Shapiro-Wilk test”.

| Variable | FC (n=17) | KD (n=59) | *P* value |
| --- | --- | --- | --- |
| Age (month) | 30.00 (19.00-38.00) | 31.00 (20.00-49.00) | 0.454 |
| Sex (male/female) | 9/8 | 26/33 | 0.711 |
| WBC (10^9^/L) | 10.60±3.77 | 13.81±4.34 | 0.007* |
| PLT (10^9^/L) | 344.65±80.31 | 347.59±117.41 | 0.923 |
| HB (g/L) | 116.35±9.60 | 109.00±11.71 | 0.021* |
| N% | 50.00 (39.00-63.00) | 69.50 (60.75-75.55) | <0.001* |
| L% | 43.00 (29.00-55.00) | 22.20 (17.40-31.60) | <0.001* |
| RBC (10^9^/L) | 4.39 (4.03-4.58) | 4.05 (3.84-4.37) | 0.073 |
| MPV (fl) | 9.50 (9.05-10.00) | 9.80 (9.40-10.35) | 0.100 |
| PDW (fl) | 10.10 (9.15-10.80) | 10.20 (9.55-11.65) | 0.295 |
| CRP ((mg/L) | 18.00 (8.00-31.00) | 48.94 (25.73-82.75) | <0.001* |
| PCT (ng/mL) | 0.23 (0.10-0.56) | 0.56 (0.22-1.21) | 0.019* |
| ALB (g/L) | 42.00 (37.30-45.00) | 38.30 (35.10-41.25) | 0.002* |
| AST (U/l) | 32.10 (27.40-37.00) | 27.40 (23.85-38.55) | 0.178 |
| ALT (U/l) | 15.30 (13.00-22.80) | 24.50 (15.45-69.75) | 0.034* |

*Note: FC, febrile controls; KD, Kawasaki disease; WBC, white blood cell counts; PLT, platelet counts; HB, hemoglobin; N%, percentage of neutrophils; L%, percentage of lymphocytes; RBC, red blood cell counts; MPV, mean platelet volume; PDW, platelet distribution width; CRP, C-reactive protein; PCT, pro-Calcitonin; ALB, albumin; AST, aspartate aminotransferase; ALT, alanine aminotransferase;* ******P < 0.05*

**Comment 8:**

-men; women

-male; female

**Response 8:**

We changed "men" and "women" into "male" and "female". Thank you.

**Comment 9:**

- serum PDGF-CC levels; with

- serum levels of; with the

**Response 9:**

According to your suggestion, we changed this sentence to “Correlation of serum levels of PDGF-CC with the general laboratory data and inflammatory cytokines in KD”.

**Comment 10:**

- Correlation between PDGF-CC and general laboratory data and inflammatory cytokines in KD was performed by Pearson correlation analysis.

- the same as above

**Response 10:**

According to your suggestion, we changed this sentence to “Correlation between serum levels of PDGF-CC and the general laboratory data and inflammatory cytokines in KD was performed by Pearson correlation analysis”.

**Comment 11:**

- Potential diagnostic value of serum PDGF-CC for KD

- why no PDGF-CC cut off diagnostic values were included in this section?

**Response 11:**

The PDGF-CC cut off diagnostic value is 2.22, we have added it to this section.

We have added the above content to the revised manuscript (paragraph 5, page 6). The relevant contents are provided below for your quick reference.

The results indicated that the AUC value of PDGF-CC was 0.796 (cut-off of 2.22, sensitivity of 74.58%, and specificity of 76.47%), which was higher than the AUC of other single markers.

**Comment 12:**

- Discussion

- Generally it is very poor, it is repetition of the results and cited texts rather than being explanation of the main study findings in depth and comparing them with previously known data

**Response 12:**

Thank you for your comments. However, we respectfully disagree with the above statements.

Our discussion section was framed by a brief description of the background of the study (paragraph 3, page 7), a summary of the main findings (paragraph 1, page 8), a detailed description of each of the main findings (paragraph 2, page 8 to paragraph 3, page 9), and the limitations of this study (paragraph 4, page 9).

In the detailed description section, we integrated the main findings of this study with previously known information. In brief, for “KD patients with higher serum levels of PDGF-CC than FC and HC”, we cited previous literature and wrote that “PDGF-CC was found to be involved in many cardiovascular diseases, plays an important role in regulating monocyte chemotaxis and stimulating the expression of MMP-9”. Furthermore, we associated PDGF-CC with KD by previously reported KD characteristics. We hypothesized that “PDGF-CC may play an important role in vascular inflammation in the acute phase of KD through inflammatory activation”; For “correlation of serum levels of PDGF-CC with the general laboratory data and inflammatory cytokines in KD”, we wrote by citing literature that “it has been reported that TNF-α, IL-1β, and INF-γ induce PDGF-CC mRNA expression in endothelial cells, and the stimulation of proinflammatory cytokines such as IL-1β and TGF-β also leads to high expression of the receptor PDGFR-αα in vascular endothelial cells and macrophages”. Similarly, we linked PDGF-CC to KD through previously reported characteristic laboratory findings and pathophysiological feature of KD, leading to speculation that “serum TNF-α, IL-1β, INF-γ, and PDGF-CC may synergistically involve KD-associated vasculitis”; For “Potential diagnostic value of serum PDGF-CC for KD”, we state the potential of PDGF-CC as a diagnostic biomarker for KD and the enhanced predictive value for KD of the combined biomarkers; For “Serum PDGF-CC and clinical laboratory parameters between the KD‑NCAL and KD‑CAL groups”, We analyzed the reasons for the lack of differences in PDGF-CC between groups. Given that there were no differences between groups in positive indicators reported in previous literature, it was speculated that the small sample size might be the reason.

Therefore, each section in our discussion is interconnected, and the absence of a certain part may leave the overall structure incomplete. We have also linked previous findings to the content of this study. Perhaps because most of the literature we cite is related to basic experiments, directly writing out the regulation manners and conclusions, and lacking detailed data descriptions, you may be confused and think we have not compared with previous literature. Accordingly, we added three clinical studies at the beginning of the discussion, linking PDGF to vasculitis. Importantly, we have also provided an in-depth explanation of the main findings, including speculation that “PDGF-CC may play an important role in vascular inflammation in the acute phase of KD through inflammatory activation”, as well as “serum TNF-α, IL-1β, INF-γ, and PDGF-CC may synergistically involve KD-associated vasculitis”.

**Comment 13:**

- Discussion section, paragraph 1. “The PDGF family……further explored the potential role of PDGF-CC in KD” .

- No need of this introductory text, rather the author should start discussing the study findings in depth

**Response 13:**

This introductory text is a brief description of the background of the study, the first part of our discussion, followed by a summary of the main findings and an in-depth discussion of the research findings.

**Comment 14:**

- Discussion section, paragraph 2. “Our study demonstrated that……for the early diagnosis of KD”.

- This text is repetition of the results rather than discussion; This is part of the conclusion rather than being discussion

**Response 14:**

Indeed, this paragraph is a summary of our main findings and conclusions and the second part of our discussion. Its existence helps to provide our main results and conclusions quickly and to discuss them in depth one by one in subsequent texts.

**Comment 15:**

-Discussion section, paragraph 3. “PDGF-CC was found to be involved in many cardiovascular diseases……through inflammatory activation” .

- A gain this is not real discussion explaining the study core findings

**Response 15:**

This paragraph is a discussion based on “The present study found that serum PDGF-CC levels were significantly higher in acute KD patients than in HC and FC”. Briefly, we cited previous literature and wrote that “PDGF-CC was found to be involved in many cardiovascular diseases, plays an important role in regulating monocyte chemotaxis and stimulating the expression of MMP-9”. Furthermore, we associated PDGF-CC with KD by previously reported KD characteristics. We speculated that “PDGF-CC may play an important role in vascular inflammation in the acute phase of KD through inflammatory activation”.

Thank you again for all your time and comments. We hope you find our revision satisfactory.

Sincerely,

Qijian Yi

-----End of Reply to Reviewer #2------

**Reply to Reviewer #3**

Dear Reviewers,

Thank you very much for taking the time to review our manuscript and for your valuable comments.

**Comment:**

Very interesting article which highlights both a little-known aspect of vascular remodeling in KD in the literature and which also seeks to use this biomarker in the diagnostic field.

There are certainly no other studies to confirm this type of use, but it is certainly an element to take into consideration

**Response:**

Yes, there are currently no other studies to confirm this type of use. We have added information in the limitations section that requires other studies to confirm our study findings.

We have added the above content to the revised manuscript (paragraph 4, page 9). The relevant contents are provided below for your quick reference.

There are several limitations to this study. First, this was a single-center study with a relatively small number of participants. Other multi-center research is necessary to confirm and extend the current results.

We hope you will be satisfied with this revised version.

Sincerely,

Qijian Yi

-----End of Reply to Reviewer #3------
